# Evaluation of lactate detection using selective multiple quantum coherence in phantoms and brain tumours

**DOI:** 10.1002/nbm.3255

**Published:** 2015-01-14

**Authors:** L M Harris, N Tunariu, C Messiou, J Hughes, T Wallace, N M DeSouza, M O Leach, G S Payne

**Affiliations:** Cancer Research UK and EPSRC Cancer Imaging Centre, Institute of Cancer Research and Royal Marsden NHS Foundation TrustSutton, Surrey, UK

**Keywords:** lactate, brain tumours, glioma, multiple quantum coherence, MRS

## Abstract

Lactate is a product of glucose metabolism. In tumour tissues, which exhibit enhanced glycolytic metabolism, lactate signals may be elevated, making lactate a potential useful tumour biomarker. Methods of lactate quantitation are complicated because of overlap between the lactate methyl doublet CH_3_ resonance and a lipid resonance at 1.3 ppm. This study presents the use of a selective homonuclear multiple quantum coherence transfer sequence (SelMQC-CSI), at 1.5 T, to better quantify lactate in the presence of lipids. Work performed on phantoms showed good lactate detection (49%) and lipid suppression (98%) efficiencies. To evaluate the method in the brain, the sequence was tested on a group of 23 patients with treated brain tumours, either glioma (*N* = 20) or secondary metastases in the brain (*N* = 3). Here it was proved to be of use in determining lactate concentrations *in vivo*. Lactate was clearly seen in SelMQC spectra of glioma, even in the presence of lipids, with high grade glioma (7.3 ± 1.9 mM, mean ± standard deviation) having higher concentrations than low grade glioma (1.9 ± 1.5 mM, *p* = 0.048). Lactate was not seen in secondary metastases in the brain. SelMQC-CSI is shown to be a useful technique for measuring lactate in tumours whose signals are otherwise contaminated by lipid. © 2015 The Authors *NMR in Biomedicine* Published by John Wiley & Sons Ltd.

## Introduction

Lactate is an end-product of glycolysis. Under normal physiological conditions it is produced only in low concentrations, but production is increased in certain pathological states, including cerebral ischemia (1), mitochondrial disease (2,3) and cancer (4–6), making it a potential biomarker in these conditions (6).

Lactate has been shown to be elevated in rodent glioma models (7). It is well known that neoplastic cells have an increased capacity for glycolytic metabolism (7) under both aerobic or anaerobic conditions as part of the malignant phenotype, thus the lactate signal would be expected to be elevated (8). Additionally, measuring the concentration of lactate in animal models has been used to monitor response to both chemotherapy and radiotherapy (9).

In the clinic, ^18^ F-fluorodeoxyglucose (^18^FDG) positron emission tomography is used to detect tumours and monitor their response to treatment by imaging their increased glucose uptake compared with normal tissue. In this situation, quantifying lactate potentially offers complementary information on (steady-state) lactate concentrations, particularly in organs such as the brain where the high glucose consumption and hence ^18^FDG uptake of normal tissue obscures their differentiation.

Measuring lactate concentration *in vivo* using ^1^H NMR is not straightforward. Lactate is an AX_3_ spin system characterized by a –CH_3_ doublet peak at 1.3 ppm, a –CH quartet at 4.1 ppm, and a *J*-coupling constant of 6.9 Hz. The quartet at 4.1 ppm is small and often affected by water suppression. Additionally, there is a large overlap between the larger signal for the lactate –CH_3_ resonance at 1.3 ppm and the –CH_2_– resonances of lipids. This frequently leads to the lactate resonance being largely obscured by lipid signals either from surrounding tissue or in some cases from within the tumour itself (10).

Several methods have been proposed to optimize lactate signal detection in the presence of lipids, mostly by exploiting the *J*-coupling of lactate. The simplest of these is to use long echo times, which will favour lactate detection over lipid (11). This method is sufficient in tissues where lipid is lower; however, it fails to eliminate strong lipid signals, such as those often found in tumours. An alternative method is to use spin-echo difference editing. This is achieved by subtracting signal from a measurement using a non-selective refocusing pulse from one with a selective refocusing pulse. The lactate signals, therefore, add while any singlets are cancelled, including signal from lipids (12,13). A significant problem with this method is that even small tissue motion leads to incomplete subtraction of the large lipid signals. There are 2D methods for lactate detection, such as *J*-resolved spectroscopy (14). These have been shown to offer good discrimination between lactate and lipid in human brain tumours (15); however, the acquisition time is long and the method suffers from motion artifacts. Another technique suggested is the use of multiple quantum filtering, which offers a compromise between detection of the lactate signal and suppression of that from lipid (16,17). This method uses a series of RF pulses and magnetic gradients to select for specific quantum coherence pathways for the lactate signal. This technique has proved to be able to detect low level lactate signals *in vivo* (18,19).

The purpose of this study was to evaluate a 2D chemical shift imaging (2D-CSI) version of this multiple quantum filtering technique (outlined by Mellon *et al*. (20)) at 1.5 T. The technique was initially tested on phantoms to determine the lactate detection and lipid suppression efficiencies of the sequence and then trialled on a group of brain tumour patients in order to determine its effectiveness in detecting lactate in this cohort.

## Experimental methods

**Figure 1:**
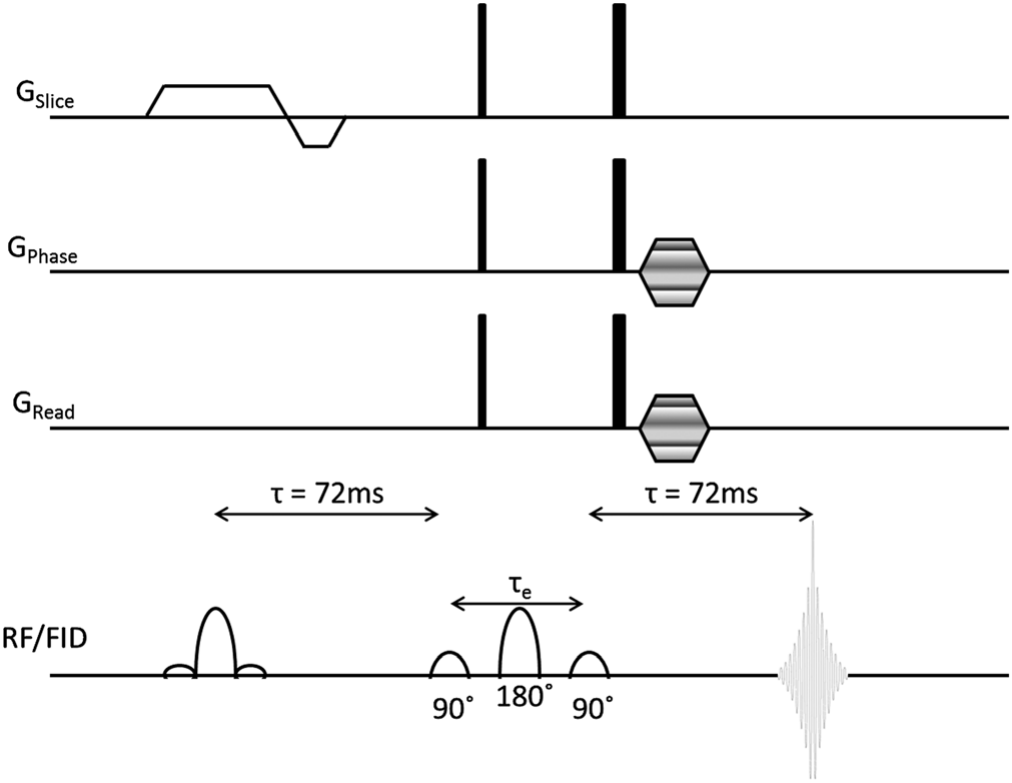
SelMQC-CSI sequence, courtesy of the group of Professor Jerry Glickson, with modifications by Dr Seung-Cheol (20). An initial slice-selective pulse is followed by a 72 ms evolution period. Following this is the multiple quantum coherence module, starting with the antiphase magnetization being split into double quantum and zero quantum coherences by a 90° frequency selective Gaussian at 4.1 ppm (CH). Coherence selection gradients are in a 1:2 ratio, followed by phase encoding in two planes for 2D-CSI measurement.

All studies were performed using a 1.5 T Siemens Avanto (Siemens Medical Systems, Erlangen, Germany). Phantom studies were performed using the standard body and phased array receiver coils, due to the large size of the phantoms. Patient studies were performed using the standard (receive only) head coil (12 elements, automatically combined by scanner software). The selective multiple quantum coherence (SelMQC)-CSI sequence (20) (Fig.1) was made available to us by Professor Jerry Glickson's group at the University of Pennsylvania (20). The main difference from the previously published sequence is the removal of the Hadamard encoding segment, so that the sequence becomes a single slice with a shorter minimum scan time. It was performed with *T*_R_ = 1500 ms, *T*_E_ = 144 ms, a 16 × 16 grid of cubic voxels (20 mm side), 20 mm slice thickness, 2 kHz bandwidth, dwell time = 0.5 ms, 1024 complex spectral points, one average, *Q*_sel_ gradient strength 26 mT/m, and acquisition time 6 m 24 s. The durations of the excitation pulse, the selective double quantum creation and detection pulses, and the refocusing pulse were 3 ms, 7 ms, and 7.6 ms respectively. The duration of the multiple quantum (MQ) mixing period was 17.5 ms. Point-resolved spectroscopy (PRESS)-localized single voxel spectroscopy (SVS) was used to collect data quickly to provide a water concentration reference (*T*_E_ = 135 ms, (20 mm)^3^ cubic voxel, *T*_R_ = 1500 ms, four averages). Institutional review board approval was granted for these additional sequence acquisitions.

### Water measurement

To determine the validity of using SVS for the water reference measurements as opposed to CSI, CSI and SVS experiments were compared. 2D PRESS-CSI (*T*_E_ = 135 ms, *T*_R_ = 1500 ms, one average, 16 × 16 grid) and SVS (*T*_E_ = 135 ms, cubic voxel, *T*_R_ = 1500 ms, four averages) experiments were performed on a spherical (diameter = 150 mm) phantom containing a solution of brain metabolites (Phantom 1) (range of voxel sizes: side 10 mm; 12 mm; 14 mm; 16 mm; 18 mm). The area under each water peak was calculated using the water referencing algorithm in the Java version of the Magnetic Resonance User Interface (jMRUI) (21), and a comparison was made between CSI and SVS across voxel sizes. Data were compared using Bland–Altman analysis (22).

### Phantom studies

A high quality (six averages) SelMQC-CSI spectrum from a cubic phantom (50 mm side) (Phantom2) containing a 20 mM lactate solution (16 × 16 grid of (35 mm)^3^ cubic voxels, 35 mm slice thickness) was collected for comparison with a single quantum lactate spectrum.

A SelMQC-CSI experiment with only one average was performed on a spherical phantom containing a solution of brain metabolites (Phantom 1) (12.5 mM N-acetylaspartate, 10 mM creatine, 5 mM lactate, 12.5 mM L-glutamic acid, 3 mM choline, 7.5 mM myo-inositol, and 50 mM KH_2_PO_4_). This was to assess how the fitting would perform on SelMQC-CSI spectra with lower signal to noise ratios, and in the presence of other metabolites.

Water reference data were collected by performing SVS experiments (*T*_E_ = 135 ms, (20 mm)^3^ cubic voxel, *T*_R_ = 1500 ms, four averages).

In order to correct for relaxation effects the longitudinal relaxation time constant (*T*_1_) and transverse relaxation time constant (*T*_2_) were measured for water and lactate in Phantoms 1 and 2. *T*_1_ was measured using a PRESS-localized saturation-recovery scheme, while *T*_2_ was measured using the same sequence with a range of echo times. Parameters were calculated using a non-linear least-squares fit between the data and the appropriate saturation-recovery and decaying exponential curves (MATLAB, MathWorks, Natick, MA, USA).

To evaluate the efficiency of lactate detection and lipid suppression, samples of lactate (20 mM) and 100% safflower oil (as in the paper by Mellon *et al*. (20)) were prepared in 15 ml spherical perspex phantoms, which were then submerged in water one at a time (Phantom 3). The SelMQC-CSI sequence and standard single quantum PRESS-CSI were performed with the same parameters. For the PRESS-CSI, the in-plane PRESS box was set to 160 mm × 160 mm, with slice thickness of 30 mm.

### Patient studies

The SelMQC-CSI sequence was performed with (20 mm)^3^ voxel size after a standard imaging investigation (including administration of contrast agent) on patients with either glioma (*N* = 20) or secondary metastases (*N* = 3) in the brain. Pathological diagnoses were glioblastoma multiforme (GBM, *n* = 14), anaplastic oligodendroglioma (*n* = 1), subependymal giant cell astrocytoma (*n* = 2), low grade glioma (LGG, *n* = 3), ungraded glioma (*n* = 1), and brain metastasis (*n* = 3). Patients had previously received either a partial resection or a total resection of the tumour. Two of the three patients with LGG had received chemotherapy. Those patients with a GBM had previously received radiotherapy with chemotherapy, but had relapsed by the time of these scans. No effect due to the presence of titanium screws was seen in any of the spectra.

Data were acquired using the standard head coil. The shim volume was placed over the tumour and shimming was performed manually. Saturation slices were placed over the scalp and ventricles. In this pilot study standard single quantum PRESS-localized single voxel spectra (*T*_E_ = 135 ms, (20 mm)^3^ cubic voxel, *T*_R_ = 1500 ms, 128 averages) were acquired to compare lactate visibility between the multiple quantum filtered and standard sequences. Single voxel data were collected rather than PRESS-localized CSI due to time constraints. Voxels were placed centrally over the tumour, avoiding resection cavities and necrosis where possible.

Water reference data were collected using an SVS experiment, as for phantom studies.

### Processing

All spectra were processed using the QUEST (Quantitation based on Quantum Estimation) package in jMRUI (21), fitting with a lactate doublet lineshape. Approximate concentrations were calculated, including correcting for *T*_1_ of water (approximately 1135 ms in brain (23)) and lactate (approximately 1250 ms in brain (24)), and for *T*_2_ of water (around 89 ms in brain (23)). Corrections were not made for the *T*_2_ relaxation time of lactate, as reported values for this have a large range (280–1200 ms in glioma) (23–26). However owing to the long *T*_2_ of lactate this correction is likely to be small. The water concentration was assumed to be 55 M within phantoms and 30 M within tumours (27).

## Results

### Phantom experiments

#### Water measurement

Standard single quantum CSI and SVS experiments performed over a range of voxel sizes (side of 10 mm, 12 mm, 14 mm, 16 mm, 18 mm) showed that the two methods offered similar values for the area under the water peak, *A* (calculated using the water referencing algorithm in jMRUI). The average value for the ratio *A*_SVS_/*A*_CSI_ was 0.93, with 95% limits of agreement of 0.91–0.96. Thus while there is a small bias (which can be included in the calculation), since SVS measurements take significantly less time, in comparison with CSI, this justified the use of SVS to measure the water reference signal in further experiments.

#### Estimated concentrations

SelMQC-CSI spectra from the cubic phantom containing 20 mM lactate solution (Phantom 2) were processed using the QUEST package in jMRUI. The measured *T*_1_ values of water and lactate were 2919 ± 89 ms and 1830 ± 26 ms, with corresponding *T*_2_ values of 1915 ± 28 ms and 942 ± 25 ms respectively (mean ± standard error, estimated from the goodness of fit to the theoretical decay curves). The calculated concentration of lactate in the phantom (including the corrections for relaxation and for the difference in signals between SVS and CSI measurements of water) was 16.3 ± 0.5 mM (fractional Cramer–Rao lower bound (CRLB) = 1 × 10^−9^), assuming a water concentration of 55 M. This result is an underestimate of 19% compared with the true value of 20 mM.

**Figure 2:**
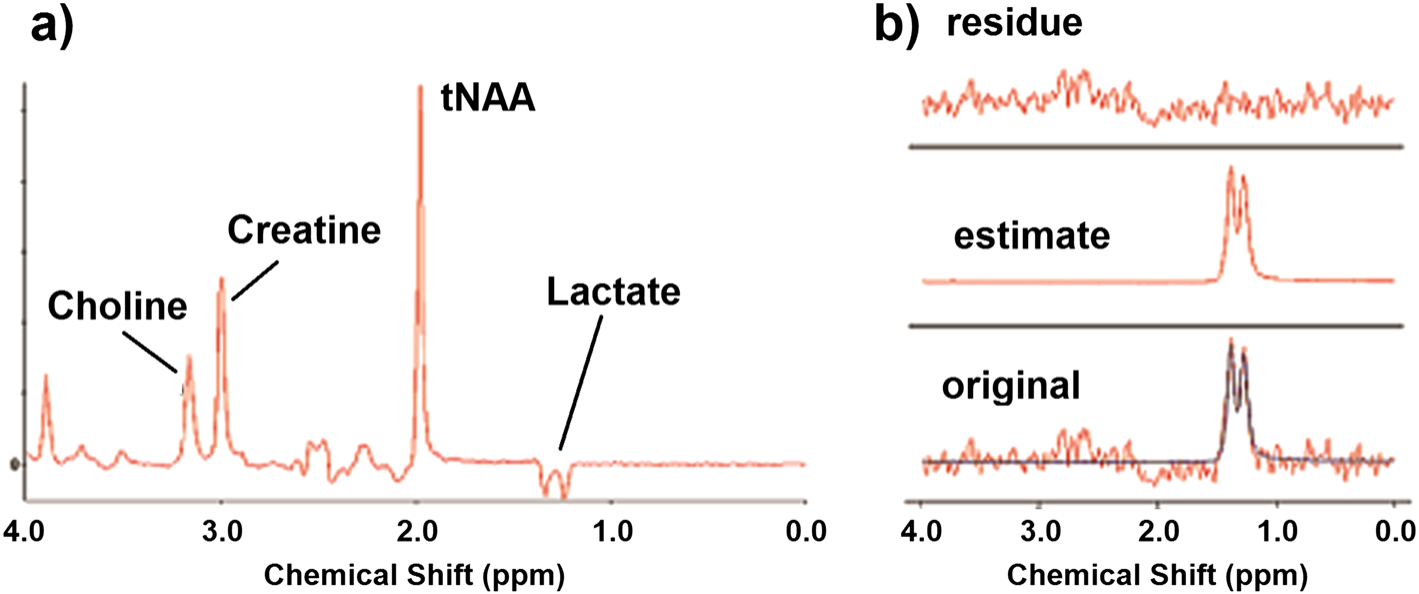
Data from Phantom 1 containing a solution of major brain metabolites (3 mM choline, 10 mM creatine, 12.5 mM glutamate, 12.5 mM NAA, 7.5 mM myo-inositol, and 5 mM lactate, plus 50 mM KH_2_PO_4_, 56 mM NaOH, 0.1% azide, and 0.1% Gd-DTPA). The phantom was 150 mm in diameter, using a 16 × 16 grid of (20 mm)^3^ voxels. All data were apodized with 1 Hz line-broadening prior to Fourier transformation and phase correction. (A) PRESS-localized single voxel spectrum (*T*_E_ = 135 ms, voxel size = (25 mm)^3^, 128 averages). (B) SelMQC-CSI spectrum fitted using jMRUI with an experimentally derived basis set. Bottom: SelMQC-CSI spectrum (red) with jMRUI model fit (blue). The peaks between 2 and 3 ppm arise from coupled spins, primarily glutamate, that are not totally eliminated by the MQ filter. Middle: estimate for the peak. Top: residual.

A SelMQC-CSI spectrum from the brain metabolite phantom (Phantom 1) was also fitted using QUEST (Fig.2). The measured values of *T*_1_ and *T*_2_ were much shorter in this phantom, with *T*_1_ values of water and lactate being 389 ± 8 ms and 636 ± 41 ms, and corresponding *T*_2_ values being 317 ± 1 ms and 437 ± 13 ms respectively. This gave a calculated concentration of 4.47 ± 0.13 mM (fractional CRLB = 0.08). The quoted concentration of lactate in the solution was 5 mM.

#### Lactate detection and lipid suppression (Phantom 3)

**Figure 3:**
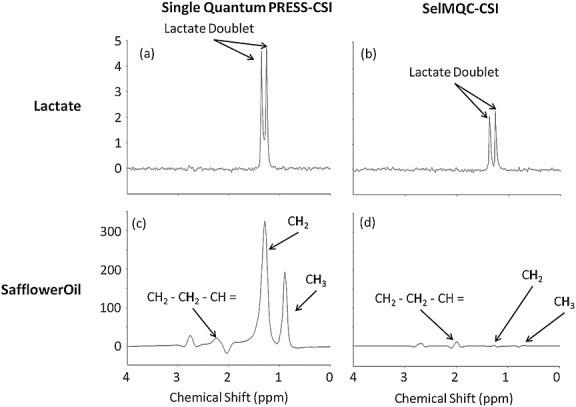
Data from 20 mM lactate solution (a), (b) and safflower oil phantoms (c), (d). (a), (c) Single quantum spectra for lactate and safflower oil respectively; (b), (d) the corresponding SelMQC-CSI spectra.

Single quantum PRESS-CSI and SelMQC-CSI spectra of lactate are shown in Figure3(a), (b). There is 49% retention of the lactate doublet peak height at 1.3 ppm, with noise levels being similar between the two sequences.

Single quantum PRESS-CSI and SelMQC-CSI spectra of safflower oil (Fig.3(c), (d)) indicated that the peak height for the lipid resonance at 1.3 ppm in the PRESS-CSI spectrum was approximately two orders of magnitude larger than that for the 20 mM lactate solution (Fig.3(c)). In the SelMQC-CSI spectrum (Fig.3(d)) there was approximately a 98% reduction in the lipid peak at 1.3 ppm. The lipid peak at 2 ppm is not significantly attenuated by the SelMQC sequence owing to its coupling with the vinyl peak at 5.3 ppm as previously observed (20); however, this is far enough removed from the lactate peak at 1.3 ppm for it not to interfere with quantification.

#### Detection threshold (Phantom 1)

The signal-to-noise ratio (SNR, calculated as peak height divided by root mean square noise) of the SelMQC-CSI lactate peak in Phantom 1 containing 20 mM lactate solution (16 × 16 grid, voxel size = (20 mm)^3^, one average) is approximately 19. This suggests that the minimum concentration of lactate detectable *in vivo* is of the order of 1 mM (i.e. for SNR > 1), with use of this coil, sequence, and parameters.

### Patients

**Figure 4:**
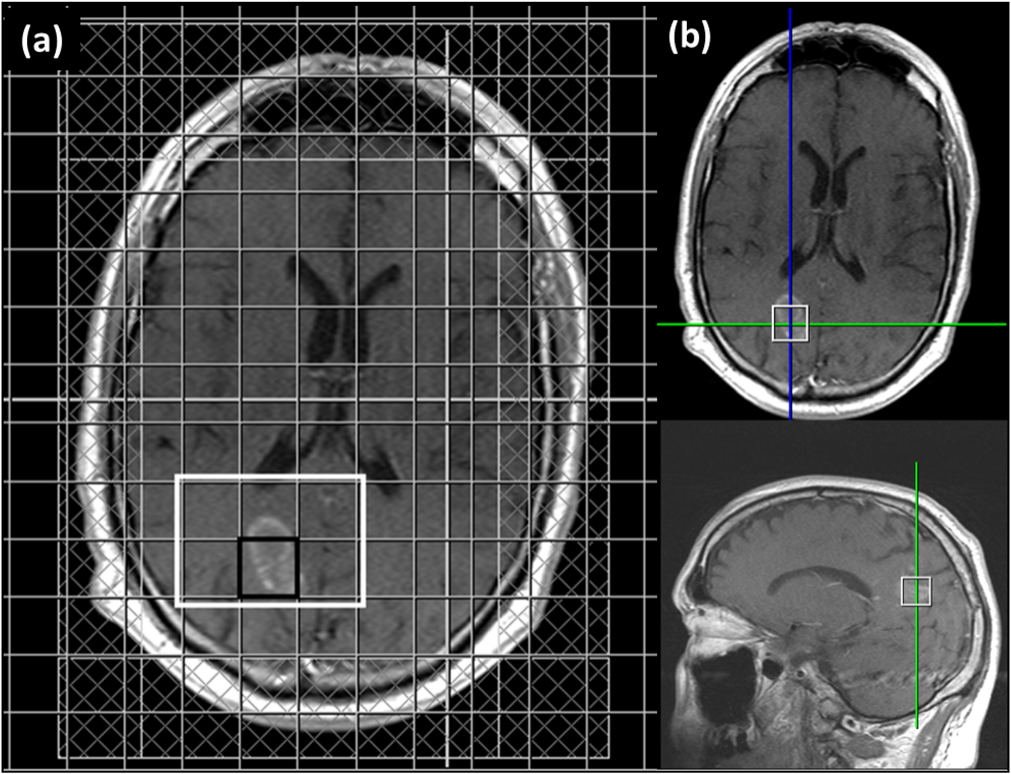
Example placement of (a) SelMQC-CSI grid and (b) corresponding PRESS voxel on *T*_1_-weighted images of patient with glioblastoma following adminstration of contrast agent. In (a) the black-edging indicates the voxel corresponding to the tumour and the white-edged region is the shim volume. Saturation slices to suppress lipid under the scalp are shown hatched. The white cross-hairs visible indicate the location of orthogonal slices currently viewed, but have no significance here.

**Figure 5:**
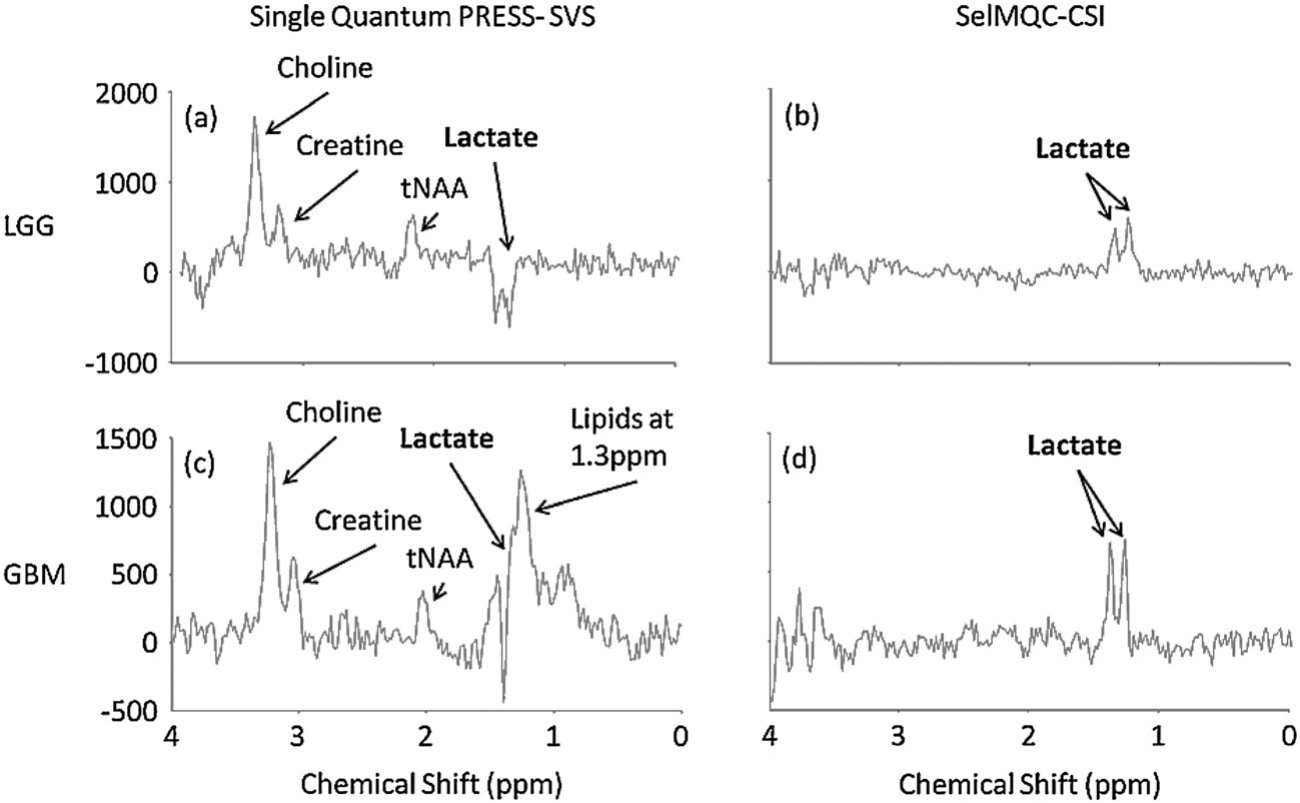
Example SVS and SelMQC-CSI data from patients with LGG (a), (b) and glioblastoma (c), (d). (a) SVS spectra for LGG show a distinct lactate doublet at 1.3 ppm, with no visible signal for lipids. (b) SelMQC-CSI data show a distinct lactate doublet. Vertical scale as (a). (c) SVS spectra for GBM show a combination of in phase lipid signal and antiphase lactate at 1.3 ppm. (d) SelMQC-CSI spectra for this patient show only the doublet for lactate. Vertical scale as (c).

An example of voxel placement in an LGG is shown in Figure4. In 15 patients with glioma the SelMQC-CSI spectrum of the tumours showed a distinct lactate signal at 1.3 ppm. Two examples are shown in Figure5: an LGG (Fig.5(b)) and a relapsed GBM (Fig.5(d)). In contrast, standard single quantum SVS spectra showed a combination of signals from both lactate and lipids (Fig.5(a), (c)). This overlap was more problematic in the relapsed GBM and anaplastic oligodendroglioma spectra, with some cases in which the lipid fully obscured the lactate signal, while in other cases it overlapped to the extent that lactate was not able to be reliably quantified. In the standard spectra, lactate could only be measured with confidence in two spectra from relapsed GBMs, whereas in the SelMQC-CSI lactate was clearly resolved in all relapsed GBMs. In the metastatic lesions there were no distinguishable peaks at 1.3 ppm in either the PRESS or the SelMQC-CSI spectra.

The SelMQC-CSI spectra were fitted in QUEST, showing significantly higher lactate in GBM (7.3 ± 1.9 mM, mean ± standard deviation) compared with LGG (1.9 ± 1.5 mM, *p* = 0.049).

## Discussion

This study has successfully shown that SelMQC-CSI provides better quantification of lactate in regions of elevated lipid signal. The sequence was tested for lactate detection efficiency, where 49% retention of the signal was recorded compared with the standard PRESS-CSI sequence. In principle the SelMQC sequence can only recover 50% of the lactate signal, due to the selection of a single coherence-transfer pathway (28), thus this value of 49% is very close to the theoretical maximum. When quantified in two different phantoms the SelMQC sequence yielded approximately the expected values, but underestimated lactate concentrations by approximately 19% and 11%. The cause of this loss is not totally clear, but it is likely to be a combination of losses in the sequence, probably including imperfect refocusing, which will affect both PRESS-CSI and SelMQC-CSI sequences. Measurements of lactate concentrations *in vivo* are also likely to suffer from this underestimate, as well as from greater uncertainty in the relaxation corrections, since it is not practicable to measure the relaxation times in each tumour (especially for the lactate resonance). Lactate peaks *in vivo* will often have a lower SNR than in the phantom, increasing the uncertainty in the fit, but this should be reflected in the CRLB estimate produced by the fitting package (jMRUI).

For future studies we suggest using MRSI measurements for the tissue water reference, since this will ensure greater consistency in measurement technique, and the possibility to estimate lactate concentrations in a greater range of voxels. A short *T*_E_ for this measurement would also reduce the dependence of the calculated concentrations on the *T*_2_ of water.

The SelMQC-CSI sequence suppresses signal from the 1.3 ppm lipid peak with a reduction of more than 98%. In order to avoid misidentifying residual lipid as lactate, the SNR of the SelMQC lactate peak must therefore be much larger than 2% of that of the 1.3 ppm lipid in the corresponding non-filtered spectrum. The estimated lipid suppression may be insufficient in some extracranial tissues, where lipid may be significantly higher. For some applications outside the brain, further optimization of lipid suppression would be required.

When the SelMQC-CSI sequence was applied in a group of previously treated brain tumours, it was shown to be able to detect lactate *in vivo* even in the presence of high lipid signals. A previous report stated that lactate is present in higher concentrations in newly diagnosed high grade glioma compared with low grade (5), in keeping with what we present here in a group of treated gliomas.

It may be noted that the Sel-MQC lactate yield in Figure5(b) appears to be more than 50% of the standard measurement in Figure5(a). There are two potential contributions to this effect. First, unresolved lipid signal may be present in (a) that still subtracts from apparent lactate signal (this is more obvious in (c)). Second, SVS lactate spectra suffer from partial volume subtraction effects (29). This reduces the total measured lactate signal from PRESS-localized single voxel spectra. For quantifying lactate using standard PRESS localization this effect needs to be taken into account (but it is not required for the work presented here).

Glioma xenograft studies have shown that glycolysis is more prominent in hypoxic areas than in diffuse infiltrative growth (30). Therefore, lactate measurements have great potential as a biomarker of response in therapies that induce hypoxia such as radiotherapy. Furthermore, evaluation of first line treatment with adjuvant radiotherapy and temozolomide is sometimes difficult owing to the phenomenon of pseudoprogression on contrast-enhanced MRI (31), which is difficult to differentiate from true progression and may be helped by including measurement of tissue lactate content. The problem of lipids interfering with lactate signal is probably greatest with high grade tumours, as lipids may also reflect severity of tissue damage (5).

## Conclusions

This study showed that the SelMQC-CSI technique offers a good means of lipid suppression while maintaining good lactate detection efficiency. Lactate was successfully measured in both phantoms and in a range of brain tumours. Prior experiments with this sequence have been performed at 3 T; this study shows that the method is also clinically useful at 1.5 T, with a minimum detection threshold of approximately 1 mM for a 6.5 min acquisition. Further study with this sequence will include expansion of the brain tumour group to study lactate in non-enhancing peritumoral regions and the evaluation of lactate in extracranial tumours.

## Abbreviations

CRLB: Cramer–Rao lower bound
CSI: chemical shift imaging
^18^FDG: ^18^F-fluorodeoxyglucose
GBM: glioblastoma multiforme
jMRUI: Java version of the Magnetic Resonance User Interface
LGG: low grade glioma
PRESS: point-resolved spectroscopy
QUEST: Quantitation based on Quantum Estimation
SelMQC: selective multiple quantum coherence
SNR: signal-to-noise ratio
SVS: single voxel spectroscopy

## References

[1] Combs DJ, Dempsey RJ, Maley M, Donaldson D, Smith C (1990). Relationship between plasma glucose, brain lactate, and intracellular pH during cerebral ischemia in gerbils. Stroke.

[2] José da Rocha A, Túlio Braga F, Carlos Martins Maia A, Jorge da Silva C, Toyama C, Pereira Pinto Gama H, Kok F, Rodrigues Gomes H (2008). Lactate detection by MRS in mitochondrial encephalopathy: optimization of technical parameters. J. Neuroimaging.

[3] Lin DDM, Crawford TO, Barker PB (2003). Proton MR spectroscopy in the diagnostic evaluation of suspected mitochondrial disease. Am. J. Neuroradiol.

[4] Blamek S, Larysz D, Ficek K, Sokol M, Miszczyk L, Tarnawski R (2010). MR spectroscopic evaluation of brain tissue damage after treatment for pediatric brain tumors. Acta. Neurochir. Suppl.

[5] Bulik M, Jancalek R, Vanicek J, Skoch A, Mechl M (2013). Potential of MR spectroscopy for assessment of glioma grading. Clin. Neurol. Neurosurg.

[6] Walenta S, Mueller-Klieser WF (2004). Lactate: mirror and motor of tumor malignancy. Semin. Radiat. Oncol.

[7] Terpstra M, High WB, Luo Y, de Graaf RA, Merkle H, Garwood M (1996). Relationships among lactate concentration, blood flow and histopathologic profiles in rat C6 glioma. NMR Biomed.

[8] Semenza GL (2008). Tumor metabolism: cancer cells give and take lactate. J. Clin. Invest.

[9] Bhujwalla ZM, Glickson JD (1996). Detection of tumor response to radiation therapy by *in vivo* proton MR spectroscopy. Int. J. Radiat. Oncol. Biol. Phys.

[10] Li X, Vigneron DB, Cha S, Graves EE, Crawford F, Chang SM, Nelson SJ (2005). Relationship of MR-derived lactate, mobile lipids, and relative blood volume for gliomas *in vivo*. Am. J. Neuroradiol.

[11] Charles-Edwards GD, Jan W, To M, Maxwell D, Keevil SF, Robinson R (2010). Non-invasive detection and quantification of human foetal brain lactate *in utero* by magnetic resonance spectroscopy. Prenat. Diagn.

[12] Rothman DL, Behar KL, Hetherington HP, Shulman RG (1984). Homonuclear ^1^H double-resonance difference spectroscopy of the rat brain *in vivo*. Proc. Natl. Acad. Sci. U. S. A.

[13] Schupp DG, Merkle H, Ellermann JM, Ke Y, Garwood M (1993). Localized detection of glioma glycolysis using edited 1H MRS. Magn. Reson. Med.

[14] Bodenhausen G, Freeman R, Turner DL (1976). Two-dimensional J-spectroscopy: proton-decoupled carbon-13 NMR. J. Chem. Phys.

[15] Thomas MA, Ryner LN, Mehta MP, Turski PA, Sorenson JA (1996). Localized 2D J-resolved 1H MR spectroscopy of human brain tumors in vivo. J. Magn. Reson. Imaging.

[16] He Q, Shkarin P, Hooley RJ, Lannin DR, Weinreb JC, Bossuyt VIJ (2007). In vivo MR spectroscopic imaging of polyunsaturated fatty acids (PUFA) in healthy and cancerous breast tissues by selective multiple-quantum coherence transfer (Sel-MQC): a preliminary study. Magn. Reson. Med.

[17] He Q, Shungu DC, van Zijl PC, Bhujwalla ZM, Glickson JD (1995). Single-scan *in vivo* lactate editing with complete lipid and water suppression by selective multiple-quantum-coherence transfer (Sel-MQC) with application to tumors. J. Magn. Reson. B.

[18] de Graaf RA, Luyten PR, Den Hollander JA, Heindel W, Bovee WMM (1993). Lactate imaging of the human brain at 1.5 T using a double-quantum filter. Magn. Reson. Med.

[19] Wilman AH, Allen PS (1995). Yield enhancement of a double-quantum filter sequence designed for the edited detection of GABA. J. Magn Reson. B.

[20] Mellon EA, Lee SC, Pickup S, Kim S, Goldstein SC, Floyd TF, Poptani H, Delikatny EJ, Reddy R, Glickson JD (2009). Detection of lactate with a Hadamard slice selected, selective multiple quantum coherence, chemical shift imaging sequence (HDMD-SelMQC-CSI) on a clinical MRI scanner: application to tumors and muscle ischemia. Magn. Reson. Med.

[21] Stefan D, Cesare FD, Andrasescu A, Popa E, Lazariev A, Vescovo E, Strbak O, Williams S, Starcuk Z, Cabanas M, Ormondt Dv, Graveron-Demilly D (2009). Quantitation of magnetic resonance spectroscopy signals: the jMRUI software package. Meas. Sci. Tech.

[22] Bland JM, Altman DG (1986). Statistical methods for assessing agreement between two methods of clinical measurement. Lancet.

[23] Isobe T, Matsumura A, Anno I, Nagatomo Y, Yoshizawa T, Itai Y, Nose T (2003). [Changes in 1H-MRS in glioma patients before and after irradiation: the significance of quantitative analysis of choline-containing compounds]. No Shinkei Geka. Neurol. Surg.

[24] Frahm J, Bruhn H, Gyngell ML, Merboldt KD, Hanicke W, Sauter R (1989). Localized proton NMR spectroscopy in different regions of the human brain *in vivo*. Relaxation times and concentrations of cerebral metabolites. Magn. Reson. Med.

[25] Cheong JL, Cady EB, Penrice J, Wyatt JS, Cox IJ, Robertson NJ (2006). Proton MR spectroscopy in neonates with perinatal cerebral hypoxic-ischemic injury: metabolite peak-area ratios, relaxation times, and absolute concentrations. Am. J. Neuroradiol.

[26] Kugel H, Roth B, Pillekamp F, Kruger K, Schulte O, von Gontard A, Benz-Bohm G (2003). Proton spectroscopic metabolite signal relaxation times in preterm infants: a prerequisite for quantitative spectroscopy in infant brain. J. Magn. Reson. Imaging.

[27] Gideon P, Rosenbaum S, Sperling B, Petersen P (1999). MR-visible brain water content in human acute stroke. Magn. Reson. Imaging.

[28] Thakur SB, Yaligar J, Koutcher JA (2009). In vivo lactate signal enhancement using binomial spectral-selective pulses in selective MQ coherence (SS-SelMQC) spectroscopy. Magn. Reson. Med.

[29] Kelley DA, Wald LL, Star-Lack JM (1999). Lactate detection at 3 T: compensating J coupling effects with BASING. J. Magn. Reson. Imaging.

[30] Hamans B, Navis AC, Wright A, Wesseling P, Heerschap A, Leenders W (2013). Multivoxel ^1^H MR spectroscopy is superior to contrast-enhanced MRI for response assessment after anti-angiogenic treatment of orthotopic human glioma xenografts and provides handles for metabolic targeting. Neuro. Oncol.

[31] Wen PY (2010). Therapy for recurrent high-grade gliomas: does continuous dose-intense temozolomide have a role?. J. Clin. Oncol.

